# Ureteroiliac Artery Fistula in a Young Woman with Short Bowel Syndrome for Radiation Enteritis

**DOI:** 10.1155/2010/287034

**Published:** 2010-03-14

**Authors:** Lidia Santarpia, Massimiliano Creta, Umberto Marcello Bracale, Roberto Ciccarelli, Fabrizio Pasanisi, Franco Contaldo, Ciro Imbimbo

**Affiliations:** ^1^Dipartimento di Medicina Clinica e Sperimentale, AOU Federico II, 80131 Napoli, Italy; ^2^Dipartimento di Urologia, AOU Federico II, 80131 Napoli, Italy; ^3^Dipartimento di Chirurgia Vascolare, Università di Palermo, 90133 Palermo, Italy; ^4^Dipartimento di Diagnostica per Immagini, AOU Federico II, 80131 Napoli, Italy

## Abstract

Ureteral-iliac artery fistula is a rare and potentially life-threatening complication, typically occurring after radiation therapy in already surgically treated cancer patients. This case report describes the diagnostic challenges and the successful management, with the positioning of an intra-arterial prosthesis, of a fistula between the internal iliac artery and the left ureter presenting as massive hematuria in a young woman with history of total colectomy and pelvic radiotherapy for rectal cancer and subsequent wide ileal resections and bilateral ureteral stent positioning for radiation enteritis. Ureteroiliac artery fistulas require a prompt diagnosis and intervention, to avoid life threatening clinical events.

## 1. Case Report

In October 2008, a 29-year-old woman was admitted as emergency to the Department of Urology with massive hematuria and left hip colic pain. 

In 2004 she underwent a total colectomy followed by adjuvant systemic chemotherapy and radiotherapy for rectal carcinoma arising on colon familial adenomatous poliposis. Between 2005 and 2007 she developed several intestinal occlusions secondary to radiation enteritis requiring four laparotomies followed by resections of aderential bridles and wide ileal segments. Finally a cutaneous ileostomy was confectioned and daily Parenteral Nutrition prescribed. In 2006 intestinal continuity was restored. In February 2008, a Computed Tomography (CT) scan showed a bilateral hydroureteronephrosis due to a nonhomogeneous mass completely filling the pelvic region and bilateral ureteral stents were placed in order to preserve renal function. In April 2008, an exploratory laparotomy was performed which identified an abscessual collection due to the dehiscence of ileoanal anastomosis: the collection was drained and a new cutaneous ileostomy confectioned. In July and in September 2008, the right and left ureteral stents were, respectively, replaced due to infection. 

**Figure 1:**
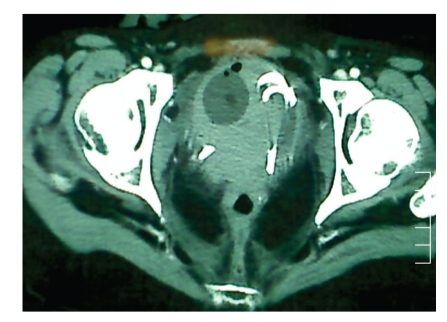
CT scan demonstrating bladder completely filled with blood clots, catheter balloon, and ureteral stents.

**Figure 2:**
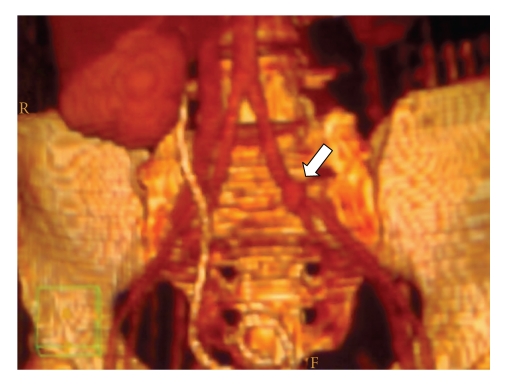
3D CT rendering showing pseudoaneurysm of left common iliac artery where it cross the left ureter (white arrow). The latter is not visible because of not functioning left kidney.

**Figure 3:**
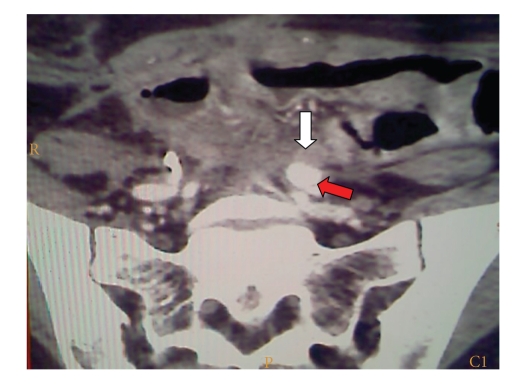
CT scan demonstrating left common iliac artery (red arrow) and left ureter (white arrow) which appear not dissociable.

On admission, a three-way catheter with a bladder washing was placed. A CT scan with contrast medium (CM-CT) showed bladder tamponade (Figure 1) and blood clots in both renal pelvis: the site of bleeding could not be identified. A cystoscopy was performed, bladder clots were evacuated, and bladder bleeding was excluded. A subsequent abdominal CM-CT scan showed left ureteral stent dislocation in the renal parenchyma with a small superior renal pole infarction. A selective left renal arteriography did not reveale primary renal bleeding. During the attempt of the left ureteral stent removal, the patient had a massive ureteral haemorrhage and collapsed. She was promptly revived with plasma expanders and blood transfusions. A ureteral catheter was positioned with a continuous bladder washing. During the ensuing week, she had intermittent but self-limiting bleeding from the left ureteral catheter. A second arteriography was unremarkable; a left superior renal pole selective artery embolization was performed but it failed to control bleeding. After one-week stabilization, a further attempt to remove the ureteral catheter was done followed by a massive ureteral bleeding. After further counseling with general and vascular surgeons for an exploratory laparotomy, a third CM-CT integrated by colour Doppler ultrasound examination was performed which identified an hydronephrotic, nonfunctioning left kidney with a left hydroureter until the cross with left common iliac artery. A pseudoaneurysm of the right proximal common iliac artery adjacent to the left ureter was detected, and an ureteroarterial fistula was suspected (Figures 2 and 3). The patient underwent to the placement of a covered, self-expandable vascular stent with angioplasty balloon without any further urinary bleeding up to now, that is, 4 months after the intervention. Moreover, a full recovery of left renal function was obtained.

## 2. Discussion

Arterioureteral fistulae occur very rarely, often difficult to indentify, like the one described in this case report, and could be associated with a high mortality rate [1] mainly due to delay in diagnosis and treatment. Radiation therapy, previous vascular surgery, prolonged ureteral stenting, prior pelvic surgery, vascular pathology, and endoureteral procedure all are conditions that can predispose to fistula formation between and artery and the ureter [2]. The patient described in this report had chronic indwelling stents and previous radiation therapy to the pelvis. The constant pulsation of the iliac artery is transmitted to a very compromised ureter (hydroureteronephrosis, infection, pelvic surgery, hypotension, and finally left hydroureter) resulting a pressure necrosis, most likely where the ureter crosses the iliac artery [3, 4]. To avoid fistula formation in debilitated patients treated previously with radiation and surgery, the use of small and soft stents is recommended [5]. 

The treatment of these fistulae has also changed overtime, from open surgery to stent-graft repair, which is less invasive than the open approach [4–6].
